# Technical challenges in the isolation and analysis of circulating tumor cells

**DOI:** 10.18632/oncotarget.11191

**Published:** 2016-08-10

**Authors:** Emma E. van der Toom, James E. Verdone, Michael A. Gorin, Kenneth J. Pienta

**Affiliations:** ^1^ The James Buchanan Brady Urological Institute and Department of Urology, Johns Hopkins University, School of Medicine, Baltimore, MD, USA

**Keywords:** circulating tumor cells, CTCs, rare cell isolation, prostate cancer

## Abstract

Increasing evidence suggests that cancer cells display dynamic molecular changes in response to systemic therapy. Circulating tumor cells (CTCs) in the peripheral blood represent a readily available source of cancer cells with which to measure this dynamic process. To date, a large number of strategies to isolate and characterize CTCs have been described. These techniques, however, each have unique limitations in their ability to sensitively and specifically detect these rare cells. In this review we focus on the technical limitations and pitfalls of the most common CTC isolation and detection strategies. Additionally, we emphasize the difficulties in correctly classifying rare cells as CTCs using common biomarkers. As for assays developed in the future, the first step must be a uniform and clear definition of the criteria for assigning an object as a CTC based on disease-specific biomarkers.

## INTRODUCTION

The estimated risk of developing cancer during one's lifetime is approximately 40%, with nearly 1 in 5 cancer patients dying as a result of their disease [1]. The metastatic cascade is a poorly understood process that begins with cell migration and intravasation into the circulation [2, 3]. Cancer cells that enter the bloodstream are termed circulating tumor cells, or CTCs. It is estimate that millions of CTCs continuously circulate throughout the body; however, it remains unclear what percentage of these cells enter the circulation through an active process *versus* passive sloughing [4–6]. CTCs that survive the physical stress of the circulation and avoid immune clearance can extravasate at distal sites. These cells, known as disseminated tumors cells (DTCs), may remain dormant for many years prior to progression to clinically-detectable metastases [7, 8].

CTCs and DTCs hold promise as functional biomarkers of the metastatic process, both for scientific inquiry and clinical applications. However, CTCs have been studied more extensively than DTCs as biomarkers of solid malignancies, partially due to the ease of sample collection [9–13]. CTC detection relies on venipuncture, rather than solid tissue biopsy or bone marrow aspiration. A major benefit of liquid-biopsy based approaches is that they can be performed repeatedly with low risk of side effects, enabling a dynamic measurement of CTCs as an indicator of disease burden and response to therapy [14–18].

The significance of CTCs as functional biomarkers of solid malignancies is evidenced by the vast array of techniques that have been developed for their detection. The goal of this narrative review is to summarize the technical limitations and pitfalls of common strategies for the isolation and analysis of CTCs. In addition, we describe the difficulty of accurately identifying cells as CTCs using only epithelial biomarkers. Because the main focus of our laboratory is prostate cancer (PCa), many of the provided examples pertain to this disease. Nevertheless, the message of this paper is applicable for most solid cancers.

## CTC ISOLATION BASICS: FINDING A “NEEDLE IN A HAYSTACK”

**Figure 1:**
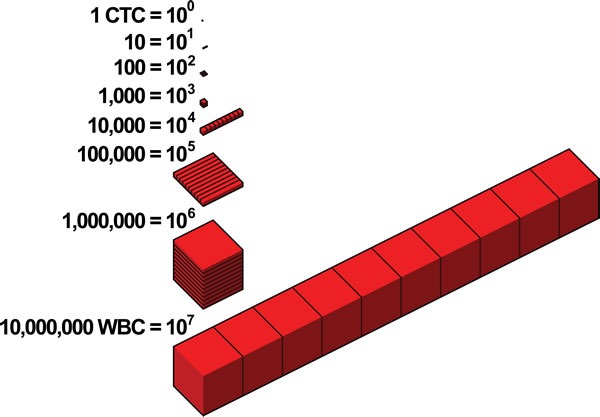
Detecting a CTC is analogous to figuratively looking for a needle in a haystack

In patients with advanced solid cancers, CTCs often occur at very low concentrations, on the order of ~1 CTC per ten million white blood cells (WBCs) in a 7.5 mL sample of blood [17, 19]. The extremely low concentration of CTCs poses a challenge for their detection and characterization, analogous to figuratively looking for a needle in a haystack (Figure 1).

In recent years, a plethora of assays have been developed for the isolation and detection of CTCs. CTC isolation strategies can be divided into three major categories: positive selection, negative selection, and selection-free.

**Positive selection**: Enrichment methods that select for cells with CTC-like properties not exhibited by other blood cell components such as WBCs. This strategy relies on the isolation of cells based on physical properties or the expression of cell surface markers that are unique to CTCs.

**Negative selection**: Depletion methods that select for and then discard objects that have WBC-like properties. This strategy relies on the removal of WBCs and other normal blood components based on physical properties or cell surface markers that are unique to non-CTCs.

**Selection-free**: High-throughput imaging and bulk methods that do not rely on positive or negative selection for the detection of CTCs or other rare cells.

## CELLULAR PROPERTIES AND CHARACTERISTICS LEVERAGED FOR CTC ISOLATION AND DETECTION

Both positive and negative selection strategies rely on differing properties and characteristics of WBCs and CTCs within the blood. These can be grouped into three main categories: physical properties, biological markers, and functional properties.

**Figure 2:**
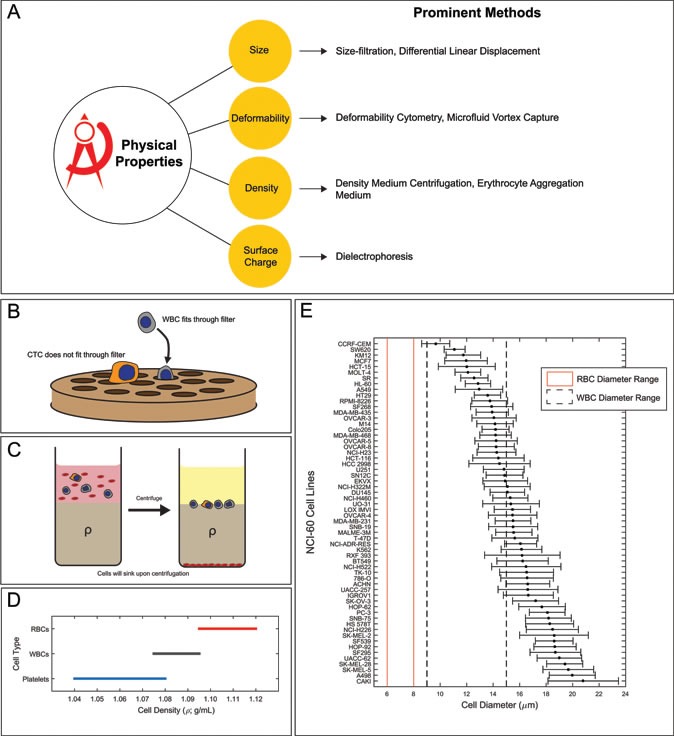
Physical properties can distinguish CTCs from other cells in the peripheral blood **A.** List of prominent methods that leverage physical properties for CTC isolation **B.** Schematic demonstrating size exclusion for depletion of white blood cells **C.** Schematic demonstrating CTC enrichment using density medium centrifugation **D.** Literature derived density ranges of major components of blood. Red: red blood cells; Gray: white blood cells; Blue: platelets **E.** Suspended cell diameter range (μm) of NCI-60 cell lines, average diameter: 15.6 μm. Vertical dashed lines indicate the range of reported WBC diameter. The vertical red lines denote the range of reported RBC diameter.

**Physical properties**: Can help distinguish CTCs from normal WBCs, these permit CTC isolation without biomarker labeling (Figure 2A).

**Biological markers**: Can help distinguish CTCs from normal WBCs and can be used to identify cells selected by other methods.

**Functional properties**: Can be used for downstream characterization of isolated CTCs.

## PHYSICAL PROPERTIES USED FOR CTC ISOLATION

**Size exclusion**: Size-based separation of CTCs relies on the fundamental assumption that epithelial-derived cancer cells are larger than other normal constituent cells of the blood (Figure 2B). This assumption, however, is based in large part from the measurement of cell lines in culture and not data on the size of actual CTCs in human circulation. Looking at data from The National Cancer Institute (NCI) 60 human tumor cell line anticancer drug discovery project, tumor cells have an average diameter of 15.6 μm (± 2.4), compared to WBCs with a range of diameters of 7-15 μm [20, 21] (Figure 2B and 2E). The pitfall, however, of using size-exclusion as a strategy for CTC isolation is the fact that many CTCs in real patient samples are close to the size of circulating WBCs. In fact, small CTCs have been associated with worse disease status [22]. Technologies for PCa CTC isolation that rely on size exclusion appear to lose anywhere from 20-50% of CTCs [23]. Kim et al. demonstrated a potential means of overcoming this issue utilizing a technology based on the selective size amplifications (SSA) of CTCs while using a multi-obstacle architecture (MOA) filter to improve both recovery rate and purity [24]. The SSA was performed by labeling CTCs with anti-EpCAM-conjugated 3 μm microbeads as a means of artificially enlarging CTC diameter, resulting in a much higher recovery and purity compared to normal size-based separation.

**Deformability**: Another physical property that has been investigated for CTC isolation is deformability. Previous studies have demonstrated that metastatic cells (from both cell lines as well as body fluids) are often more deformable than cells of lower aggressive potential [25–27]. A recent study by Bagnall et al. compared the deformability of CTCs to that of normal blood cells [28]. They measured the deformability by the length of the time required for both cell types to pass through a microfluidic device. Their study demonstrated that differences in deformability between WBCs and cancer cells are greater than changes between cancer cells of differing levels of aggression. These data suggest that differential deformability could be used to separate cancer cells from WBCs. Despite this evidence, CTCs from a subset of metastatic PCa patients in the same study were more mechanically similar to blood cells/leukocytes than to typical tumor cell lines.

An example of a technology that uses both size exclusion and deformability to capture and characterize CTCs has been developed by Celsee Diagnostics [29, 30]. This system contains a parallel network of fluidic channels with 56,320 capture chambers. Larger cancer cells are trapped and isolated in the chambers, whereas smaller blood cells, such as red blood cells (RBCs) and most WBCs, escape. A pitfall of this method is the chance of losing small sized CTCs. The system facilitates rapid capture of CTCs in the microchannel device and can also be used for downstream characterization of the captured cells by immunocytochemistry as well as DNA or RNA *fluorescence in-situ hybridization* (FISH). A benefit of this system is that it captures cells without labeling, so it is possible to use a variety of antibodies to further characterize captured cells. In a comparative study with the CellSearch system, CTC counts were significantly higher using the Celsee system demonstrating greater sensitivity for CTC detection [30].

**Density**: For the density ranges between 1.1020 - 1.1040 g/mL there is separation of most WBCs and CTCs from anucleated cells (platelets and RBCs; Figure 2C-2D). A major limitation of this type of enrichment strategy is that very small CTCs may be as dense, or even denser, than RBCs and could be lost with low-density separation media. There is emerging evidence to suggest that these small cells are of an aggressive phenotype [22].

**Surface charge**: Differences in surface charge and polarizability enables the isolation of minimally modified CTCs for future analysis. This method relies on the assumption that cancer cells have a more negative surface charge, or zeta-potential, compared to WBCs. A pitfall of this method of CTC isolation is that there is an overlap in the zeta-potential distribution, leading to WBC contamination in CTC-enriched samples. A prominent example of a CTC technology that allows for the isolation of CTCs on the basis of surface charge is the ApoStream device (ApoCell, Houston, TX) [31]. Poklepovic et al. demonstrated that in patients with metastatic PCa this system could isolate a greater number of CTCs compared to the CellSearch test [32].

## POSITIVE SELECTION STRATEGY: ENRICHMENT METHODS WITH CELL SURFACE BIOMARKERS

Isolation of CTCs using enrichment methods may rely on physical properties and/or cell surface markers. Commonly, the epithelial cell surface antigen EpCAM is used to enrich for epithelial CTCs. CTCs can also be positively enriched for using an anti-mesenchymal antibody, e.g. N-cadherin.

### Magnetic bead separation using epithelial lineage markers

**Figure 3:**
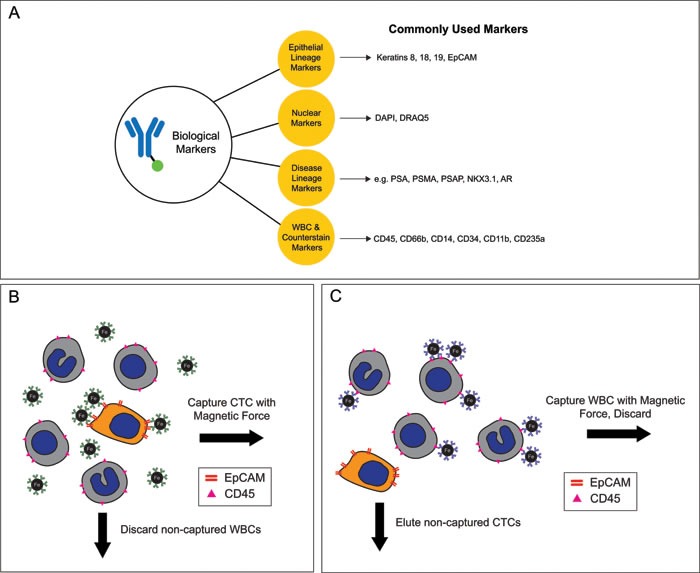
Biological markers can distinguish CTCs from other cells in the peripheral blood **A.** List of commonly used biological markers to isolate and validate CTCs **B.** Illustration of magnetic bead antigen positive selection **C.** Illustration of magnetic bead antigen negative selection.

A frequently used method for CTC enrichment with epithelial lineage markers is magnetic bead separation, where antibody-labeled ferroparticles capture CTCs in a magnetic field (Figure 3B).

**CellSearch**: The most widely used magnetic-bead based selection assay for CTC detection is the CellSearch system [12, 13, 33]. With this test, putative CTCs are positively selected on the basis of EpCAM expression and are enumerated based on positivity for cytokeratins and lack of the WBC marker CD45 [34]. Using the CellSearch system, de Bono et al. demonstrated that patients with metastatic castration resistant PCa who had fewer than 5 CTCs had better overall survival than patients with 5 or greater cells (21.7 vs 11.5 months) [12]. Furthermore, receiver operating curve analysis showed CTC count to be more predictive of overall survival than PSA reduction up to 20 weeks after initiation of therapy. A major pitfall of this system is that CTC populations are largely heterogeneous and some CTCs do not express EpCAM. Another limitation of this system is that captured CTCs lose their viability after fixation, so it is not possible to culture the collected cells or use them in functional studies [35].

**AdnaTest**: Another CTC detection method that relies on positive selection is the AdnaTest [36]. This test combines immunomagnetic enrichment of epithelial cells by using antibodies against EpCAM with a polymerase chain reaction for disease specific transcripts. For PCa, the test includes primers for prostate-specific antigen (PSA), prostate-specific membrane antigen (PSMA) and the epidermal growth factor receptor (EGFR). If one or more of these transcripts are detected, the sample is said to be positive for CTCs [36].

This test has been modified to detect the androgen-receptor splice variant V7 (AR-V7) in samples enriched for CTCs from patients with metastatic castration resistant PCa [37]. The androgen-receptor isoform encoded by splice variant 7 lacks the ligand-binding domain but remains constitutively active as a transcription factor. Presence of the AR-V7 splice variant leads to overexpression of AR-regulated genes [38]. Antonarakis and coworkers found that the presence of AR-V7 positive CTCs is highly associated with worse cancer outcomes and resistance to abiraterone and enzalutamide [37]. The authors concluded that patients with AR-V7 positive CTCs would benefit from non-AR targeted therapy. This has since been corroborated by Onstenk et al. who showed that response to cabazitaxel, a taxane chemotherapeutic agent, seems to be independent of the AR-V7 status of CTCs [39].

### Non-magnetic antigen selection using epithelial lineage markers

An alternative way to positively select CTCs on the basis of cell surface markers is with the use of microfluidic devices. There are many reports of microfluidic devices across a wide variety of cancers [40–42].

One notable example is the ‘CTC-Chip’, which consists of 78,000 microposts coated with antibodies against EpCAM. As the blood flows through the microfluidic chip, EpCAM-expressing CTCs are captured as they come into contact with the microposts [43]. A new generation of the CTC-Chip has been described which contains microfluidic channels in a herringbone pattern [44, 45]. This pattern of microgrooves induces the formation of microvortices, increasing the contact time between the anti-EpCAM antibody coated walls of the channel and the cancer cells. Captured CTCs are then stained, imaged, and directly analyzed on the device. An advantage of these chips is the fact that many different CTC specific antigens can be used for cell capture.

## NEGATIVE SELECTION STRATEGY: DEPLETION METHODS WITH CELL SURFACE BIOMARKERS

An alternative method for the enrichment of CTCs is the depletion of WBCs using antibodies against biomarkers such as CD45 and/or CD66b (Figure 3C). One pitfall of this strategy is that not all nucleated cells in the circulation are positive for CD45/CD66b. For example, endothelial cells are present in the blood of healthy persons and are CD45 negative [46]. Perhaps an even more significant limitation of negative selection is the high risk of CTC loss due to non-specific bulk effect (i.e. the loss of rare CTCs caught in massive movement of concentrated WBCs).

A common method for WBC depletion is with the use of CD45 antibodies bound to magnetic beads. One example of this is the EasySep Depletion Kit from StemCell Technologies (Vancouver, Canada). With this kit, WBCs are depleted after placing the sample in a magnetic field [46]. Similar kits are also available from Miltenyi Biotec (Bergisch Gladbach, Germany) and ThermoFisher Scientific (Waltham, Massachusetts) [47, 48].

Methods for WBC depletion are not solely limited to immunomagnetic selection. One notable example is the RosetteSep method from StemCell Technologies (Vancouver, Canada) [49]. This technique combines density gradient separation with an antibody-mediated enrichment step. Enrichment is done through negative selection and unwanted cells are targeted for depletion with tetrameric antibody complexes recognizing CD45 and CD66b on WBCs, and glycophorin A on RBCs. After density gradient centrifugation, the CD45/CD66b positive cells accumulate in the lower compartment and the CD45/CD66b-negative mononuclear cells and CTCs are present as an enriched population at the interface between the plasma and the density medium [50, 51].

## SELECTION-FREE STRATEGY: HIGH-THROUGHPUT IMAGING AND BULK METHODS FOR CTC DETECTION

It is now understood that CTCs can express EpCAM at varying levels [52–55]. This includes both cells with an epithelial and mesenchymal phenotype. This has led to the development of selection-free techniques for CTC identification. These methods include flow cytometry, high-throughput microscopy, and reverse transcription polymerase chain reaction (RT-PCR). The advantage of these methods is that there is no loss of CTCs by a selection step. However, there are limitations to these methods including the reliance on imperfect biological markers to differentiate CTCs from normal WBCs.

**Flow cytometry**: Flow cytometry was one of the first techniques used for the detection of CTCs in whole blood. A study of Gross et al. described a flow cytometric assay for the detection of rare cancer cells in blood and bone marrow by using multiple markers, each labeled by a different fluorophore [19]. With this method, the authors were able to detect as few as one cancer cell in 10^7^ nucleated blood cells. A pitfall of flow cytometric methods is that cancer cells can easily settle and/or clump throughout the process [56]. Furthermore, flow cytometry requires cells to be constituted in a single cell suspension, destroying relevant biological information associated with CTC clusters. Despite the processing throughput of high-speed sorters, this rate is less than a few thousand cells per second. As experiments typically require very large numbers of isolated CTCs, even high-speed sorters need to run for long durations. This is not only time-consuming and expensive, but may also cause cell viability issues, because the cells sorted from such long runs may no longer be usable for further characterization [57].

**High-throughput microscopy**: Examples of assays that rely on high-throughput microscopy include immunofluorescence and DNA/RNA FISH.

An important premise underlying the shift toward selection-free methods and more specifically high-throughput microscopy, is that these techniques leave no cell behind. These technologies enable screening of tens to hundreds of millions of cells without the loss of CTCs by marker selection, resulting in high sensitivity assays.

An example of a selection-free technology utilizing high-throughput imaging is the Epic Sciences platform. After lysis of the red blood cells of a patient sample, nucleated cells are plated on positively-charged proprietary adhesion slides, subjected to immunofluorescence staining and analyzed by special fluorescent scanners [58]. These scanning instruments use fiber optic array scanning technology (FAST) that can locate occult tumor cells at a rate 500 times faster than automatic digital microscopy (ADM), with comparable sensitivity and improved specificity. The exposure time is reduced by using a laser source for higher illumination levels. Another key innovation of this optical system is the exceptionally large field of view (50 × 2 mm) without a loss of collection efficiency. By collecting the fluorescence in an array of optical fibers that forms a wide collection aperture, the FAST cytometer has a 100-fold increase of view over ADM. A recent study demonstrated the analytical validity of this platform [59] and with it investigators have consistently observed a higher number of recovered CTCs relative to the CellSearch system [60].

Using the Epic platform, researchers at Memorial Sloan Kettering Cancer Center have successfully detected CTCs in PCa patients with a neuroendocrine phenotype [61], an aggressive pathologic subtype associated with resistance to hormonal therapies [62, 63]. Notably, in a direct comparison, the authors found that 6 of 13 patients with neuroendocrine or atypical castrate resistant PCa (CRPC) had fewer than 5 CTCs/7.5 mL of blood by CellSearch (5/13 had 0 cells by CellSearch) compared to Epic, where all samples had detectable CTCs (all ≥ 5 CTCs/7.5 mL) [61]. Recently, this same group applied the Epic platform to test for androgen receptor variant 7 (AR-V7) in CTCs of men with metastatic CRPC [64]. Their data was largely consistent with that of Antonarakis et al. [37] and demonstrated that men with AR-V7 positive CTCs had shorter radiographic progression-free survival and worse overall survival than men with AR-V7 negative CTCs while treated with the androgen receptor signaling inhibitors abiraterone and enzalutamide [64].

Similar to the platform developed by Epic Sciences, the Rarecyte CyteFinder system (Seattle, WA) is a novel selection-free high-throughput imaging system to detect CTCs [65–67]. This method also involves spreading nucleated cells on positively charged slides and subjects them to immunofluorescence staining. High-throughput imaging is then used to enumerate CTCs. This system also includes a retrieval device, known as the CytePicker, that allows for the isolation of single cells that can then be used in downstream molecular assays [65]. Recent data demonstrated the feasibility of 6-color immunofluorescence staining, allowing for broader phenotypic analysis of identified cells [67].

Reverse transcription polymerase chain reaction (RT-PCR); RT-PCR is a frequently used bulk method for CTC detection and characterization. Different studies suggest that the detection of CTCs with this method is more sensitive than immunohistochemistry [68, 69]. One limitation of current approaches using RT-PCR is that CTC number can only be estimated due to the fact that gene expression levels vary across CTCs [70, 71]. The recent advent of droplet digital PCR (ddPCR) represents an improvement in this technology in that it permits the detection and absolute quantification of low abundance targets in shorter times, without requiring a large number of replicates [72–74]. ddPCR is based on water-oil emulsion droplet technology. A sample is fractionated into 20,000 droplets, and PCR amplification of the template molecules occurs in every individual droplet [74]. Compared to other available digital PCR systems, this technique has a smaller sample requirement, thereby reducing costs and preserving precious samples.

## BIOLOGICAL MARKERS USED FOR CTC DETECTION

There is no consensus ‘best’ marker to define a CTC. An ideal CTC marker is expressed on every CTC, but not on the other cells in the blood sample (i.e. leukocytes, hematopoietic stem cells, endothelial cells, mesenchymal cells) and maintains expression throughout the progression of the disease (Figure 3A). Listed below are important marker categories.

**Nuclear markers**: A commonly used stain to denote the cell nucleus is DAPI (4′,6-diamidino-2-phenylindole), a fluorescent stain that binds to A-T rich regions in DNA. It can pass through an intact cell membrane, so it can be used to stain both live and fixed cells, but has a better staining pattern for fixed cells [75]. Another nuclear marker with a high affinity for DNA is DRAQ5. DRAQ5 can stain both fixed and living cells, but has a higher capacity to rapidly enter living cells [76].

**Counterstain markers**: A counterstain panel is used to demarcate cells other than CTCs, including RBCs, WBCs, endothelial cells, and hematopoietic stem cells. An example of a RBC marker is glycophorin A. This marker, however, is infrequently used as RBCs are usually lysed or removed with density centrifugation during CTC isolation [77]. In terms of identifying WBCs, CD45 is the most commonly utilized marker. Other potentially useful counterstain markers include CD66b (an activation marker for human granulocytes), CD34 (a cell surface glycoprotein selectively expressed within the human hematopoietic system on stem and progenitor cells, and also in vascular endothelial cells), CD11b, and CD14 (both expressed on macrophages) [78–81]. A list of commonly used biomarkers against constituent non-CTC cells in blood is shown in Table 1.

**Table 1:** Commonly used counterstain surface antigens for non-CTC components of peripheral blood

Component of Peripheral Blood	Cell Frequency (x 10^6^ cells/mL)	Key Surface Biomarkers
Red blood cells	3800-6200	CD235a
Platelets	140-450	CD41, CD61, CD62
Lymphocytes	1.1-3.5	CD3, CD4, CD8, CD19, CD20, CD56
Granulocytes	3.9-6.5	CD11b, CD14, CD33, CD45, CD66b, CD163, CD206
Hematopoietic stem and progenitor cells	0.001-0.007	CD34, CD45
Endothelial cells	-	CD34, CD146

**Epithelial lineage markers**: The two most often used epithelial markers are EpCAM and cytokeratins. They are used to differentiate cells of epithelial origin from hematopoietic cells. These markers form the basis of most CTC assays [34].

**Disease specific markers**: Ideally, tumor-specific markers are expressed in much higher levels in cancer cells compared with normal cells [82]. However, it has been shown that dedifferentiation and consequent loss of tissue specific markers occurs in the most aggressive cancers that would have CTCs [52]. In PCa, the identification of these markers has been problematic. Examples of potential prostate lineage makers include PSA, PSMA, PSAP, NKX3.1, and AR.

## FUNCTIONAL PROPERTIES USED FOR CTC CONFIRMATION

Once CTCs are isolated using technologies relying on physical properties and/or biological markers, further characterization using functional assays can be performed. Two notable *in vitro* assays have been described for this purpose. The first assay (Metastasis-Initiating-Cells (MIC) assay) tests the ability of CTCs to invade and digest a fluorescently labeled cell adhesion matrix [83]. The second is the EPISPOT assay, which detects specific proteins secreted during the *in vitro* culture of CTCs [84]. Furthermore, important *in vivo* information can be achieved by xenotransplantation models, by which patient-derived CTCs are injected into immune-compromised mice, after which metastases develop [85].

## CURRENT LIMITATIONS OF CTC DETECTION BASED ON ANTIBODY-BASED APPROACHES

**Figure 4:**
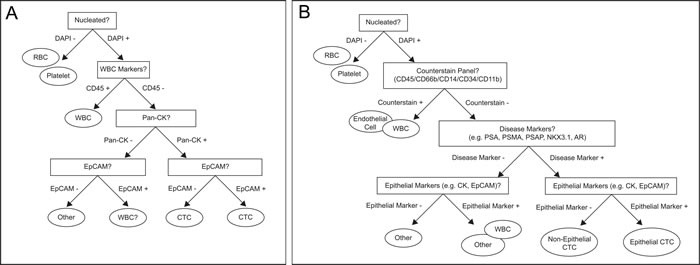
Decision trees enable consensus definitions for CTC classification using current biomarkers **A.** Decision tree for assigning a cell as a CTC, based on epithelial markers **B.** Decision tree for assigning a cell as a CTC, based on disease specific markers.

CTC technologies uniformly use biomarkers for the identification and enumeration of candidate CTCs. In the framework of the CellSearch system, the consensus biomarker set for defining a cell as a true CTC relies on nuclear, epithelial, and hematopoietic markers. To help visualize the biomarker criteria for defining a cell as a CTC under the CellSearch paradigm, we have produced a decision tree (Figure 4A).

Despite the clear benefit to immunological staining with epithelial biomarkers, several studies have shown lack of specificity and sensitivity when using EpCAM as a biological marker for CTC detection. Notably, EpCAM is non-specifically expressed on normal epithelial cells in the circulation, for example in patients with benign colon disease [86], and it may exhibit reduced expression or even be absent in cancer cells that have undergo an epithelial-to-mesenchymal transition. This may cause a false negative result [52]. Furthermore, EpCAM is expressed on M2 polarized macrophages, for example, a subset of immune cells associated with a cancer phenotype [87]. Some, but not all CTC assays rely on discarding the first volume of blood drawn to avoid skin epithelial contamination, thereby lowering the risk of false positives [88]. The data above demonstrates the low specificity of epithelial markers for CTC detection. Taken together, these data suggest the need for a disease-specific markers.

Therefore, we propose a modified decision tree to include a disease specific marker panel to confirm if a cell is a true CTC. Additionally, to decrease the CTC false discovery rate we propose utilizing an extended counterstain panel that includes CD45, CD66b, CD34, CD11b, and CD14. The first two steps in the decision tree stay the same. Next, we suggest assaying for disease specific markers (e.g. PSA PSMA, PSAP, NKX3.1, and AR). If positive, the cell is likely a CTC. If negative, it could be for example a WBC or endothelial cell. Lastly, the cell is checked for the epithelial marker pan-cytokeratin. By using a panel of disease specific markers, the assay can avoid the heterogeneity of assessing patients at varying points in treatment and progression (Figure 4B) [89].

## CONCLUSIONS

As CTCs often occur in very low concentrations, they are challenging to detect and characterize, analogous to figuratively looking for a needle in a haystack. In this review, we focus on the technical limitations and pitfalls of the most common CTC isolation and detection strategies. The presented framework aims to classify these CTC assays into different categories, based on positive selection, negative selection, and selection-free strategies. Most prominent CTC detection technologies rely on a combination of these strategies, leveraging physical properties as well as biomarkers. Furthermore we aimed to emphasize the difficulties in correctly classifying CTCs using epithelial biomarkers. The use of multiple biomarkers is usually a requirement for rare cell detection. An ideal CTC marker is expressed on every CTC, but not on the other cells in the blood and maintains expression throughout the progression of the disease. With this in mind, the first step must be a uniform and clear definition of the criteria for assigning an object as a CTC, based on disease-specific biomarkers. All told, this work will be helpful to describe the high number of different assays in this important field of translational cancer research.
